# Inflammation and Oxidative Stress in Snakebite Envenomation: A Brief Descriptive Review and Clinical Implications

**DOI:** 10.3390/toxins14110802

**Published:** 2022-11-18

**Authors:** Dabor Resiere, Hossein Mehdaoui, Remi Neviere

**Affiliations:** 1 Cardiovascular Research Team EA7525, University of the French West Indies, 97157 Fort de France, France; 2 Department of Critical Care Medicine, Toxicology and Emergency, CHU Martinique, University Hospital of Martinique, 97200 Fort de France, France

**Keywords:** snakebite, venom, elapids, viperids, oxidative stress, phospholipases A2, metalloproteinases, three-finger cardiotoxins, L-amino acid oxidase, antioxidant

## Abstract

Snakebite envenoming is a pathological condition which may occur in response to the injection of venom. Snake venoms contain a complex mixture of biologically active molecules which are responsible for a broad spectrum of clinical manifestations, ranging from local tissue injuries to fatal complications. Snake venom administration commonly provokes local tissue injury often associated with systemic effects, including neurotoxic and cardiotoxic manifestations, bleeding, acute kidney injury, and rhabdomyolysis. An important spectrum of pathogenesis of snake envenomation is the generation of reactive oxygen species (ROS), which can directly provoke tissue damage and also potentiate the deleterious consequences of inflammation at the bite site. Snake venom components known to induce oxidative stress include phospholipases A_2_, metalloproteinases, three-finger toxins, and L-amino acid oxidase. Clear evidence is mounting suggesting that inflammation and oxidative stress participate in the destructive effects of envenoming, including acute renal failure, tissue necrosis, and unusual susceptibility to bleed (hemorrhage), mostly due to hypocoagulability, neuro/cardio toxicity, and myonecrosis. Impaired regulation of oxidative stress may also set the stage for secondary/long-term complications of snakebite envenomation such as musculoskeletal disabilities. Some aspects of natural antioxidant therapeutic options are discussed in this review.

## 1. Introduction

Snakebite envenoming is the result of the injection in the body of venom, a mixture of different toxins. Annually, almost five million people are bitten by a snake, leading to 2.7 million cases of envenoming with 81,000 to 138,000 fatalities [1,2,3]. In addition, snakebite envenoming can also cause critical medical issues, including amputations and other permanent debilities, leaving people with permanent physical and psychological sequelae [1,2,3]. Snakebite envenoming typically occurs in rural poor populations in tropical and sub-tropical regions, with the highest repartition of venomous snakes being located in Africa, the Middle East, Asia, and South and Central America. In these areas, snakebite has a considerable socioeconomic impact on rural populations, because it disproportionately affects people of the working population living in less-developed countries [1]. The standard treatment for snake envenomation is antivenom, which is produced from the hyperimmunization of animals with snake toxins [1,2,3].

## 2. Pathophysiology of Snakebite Envenoming

### 2.1. Snake Families and Venom Composition

*Elapidae* (elapids) is a family of venomous snakes including coral snakes (*Micrurus*), cobras (*Naja*), kraits (*Bungarus*), and mambas (*Dendroaspis*). Elapids typically elicit neurological manifestations, such as neuromuscular blockade without major local damage [1,4]. *Viperidae* (viperids) is a family of snakes including European, Asiatic, and African vipers, including the Gaboon adder (*Bitis gabonica*), the puff adder (*Bitis arietans*), and the saw-scaled viper (*Echis carinatus*). In Central and South America, the pit viper *Crotalus* (rattlesnakes), *Lachesis* (bushmasters), and *Bothrops* genera are very common. In the United States, venomous snakes are mainly pit vipers (rattlesnakes, cottonmouths, and copperheads). 

Injection of snake venom provokes local tissue damage often associated with systemic effects, including neurotoxic and cardiotoxic manifestations, bleeding, acute kidney injury, and rhabdomyolysis [1,2,3]. The initial presenting symptoms following bites of Elapids typically associate moderate pain at the bite site and little to no local tissue damage, nausea, vomiting, vertigo, and abdominal discomfort. Neurological toxicity may include progressive motor deficits involving the cranial nerves, i.e., ptosis, dysarthria, and dysphagia, as well as descending paralysis [1,2,3]. Viperids are the most common snakes responsible for human envenomation, causing extensive local damage and systemic manifestations, such as bleeding or thrombosis, coagulopathies, and hypovolemic shock [1,2,3,4,5]. The variable biochemical and toxicological profile of venom composition determines a wide range of clinical manifestations. Snake venom metalloproteinase (SVMP), serine proteinase (SVSP) inhibitors, and phospholipase A_2_ (PLA_2_) are widely present in snake venoms of elapids and viperids. Three-finger cardiotoxins and neurotoxins (3FTx) are abundant components in elapid venoms. Cardiotoxins and neurotoxins are typically found in viperid venoms [1,2,3,6]. 

### 2.2. Mechanisms of Action

SVMP and SVSP affect aggregation function of platelets, coagulation of the blood, fibrinolysis, and the complement system [1,6,7,8]. In addition, such venom proteases display pro-coagulant or anticoagulant properties owing their interaction with many proteins of the coagulation and fibrinolytic pathways. The procoagulant effects of proteases are mainly related to specific proteolytic activation of coagulation factors and non-specific proteolysis of blood factors. PLA2s exhibit a high degree of toxic activities, on pre- or post-synaptic sites, skeletal and cardiac muscles, coagulation factors, platelet aggregation, as well as hemorrhagic, hemolytic, cytolytic, and muscarinic inhibitor activities [1,2,3,4,6,7,8]. The best-known representatives of 3FTx are α-neurotoxins. 3FTx α-neurotoxins display a high affinity for the nicotinic cholinergic receptors located at the region of the muscle fibers where the motor nerves terminate. Following the binding of the motor endplate of skeletal muscle fibers, α-neurotoxins are responsible for neuromuscular descending paralysis, a main characteristic of envenoming by many elapid species [1,6,8]. The group of 3FTxs cardiotoxins plays an important role in envenoming by Naja species and cobra elapids, eliciting plasma membrane damage, myonecrosis, and cytotoxicity. In addition, a large number of studies have demonstrated the participation of other venom components such as L-amino acid oxidase, C-type lectin-like proteins, disintegrins, and cysteine-rich secretory proteins (CRISPs), although their possible toxic roles have not been formerly demonstrated [1,6,8]. 

Other components with high toxicity but with low abundance include dendrotoxins and sarafotoxins, which are characteristic of mamba and Atractaspis venoms, respectively [8]. Dendrotoxins are a class of presynaptic neurotoxins present in mamba snake venoms (Dendroaspis), blocking voltage-gated potassium channels at the presynaptic nerve terminal, thereby facilitating the release of acetylcholine at neuromuscular junctions [6]. Sarafotoxins share a very high structural and functional homology with endothelin, activate endothelin receptors and are highly lethal and cause cardiac arrest within minutes after intravenous administration [6]. 

## 3. Inflammation in Snakebite Envenomation

### 3.1. Inflammation—General Presentation

Inflammation is an integrated coordinated process which involves the release of cytokines and chemokines by macrophages and neutrophils stimulating the migration of other leukocytes along the vascular endothelium into the inflamed tissue [9,10,11]. The immune response is needed to eliminate potential threats from the environment in order to maintain equilibrium in living organisms, preventing excessive damage to healthy cells and tissues. The mammalian immune system can be stimulated by two types of receptor systems. The germline-encoded pattern recognition receptors (PRRs) initiate the inducible innate immune response. The antigen-specific receptors generated through gene rearrangement after antigen encounters initiate the adaptive immune response [9,10,11]. A complementary host defense system that occurs independently of both PRR-based immunity and antigen-specific receptors is the so-called constitutive innate immune response. The constitutive innate immune response involves mechanisms such as chemical and physical barriers of the body, along with a wide variety of active biomolecules such as restriction factors, antimicrobial peptides, as well as mechanisms that initiate autophagy, apoptosis, and proteasomal degradation [12].

### 3.2. The Role of Constitutive Immune Responses in Snakebite Envenoming

Constitutive innate immunity establishes the first line of defense against injuries [12]. As the initial site of interaction of venoms, the dermis can take part of the constitutive innate immune response which provides protection through physical, chemical, and cellular defense mechanisms. The dermis can be considered as a physical barrier able to produce chemical substances and enzymes, while resident and infiltrating immune cells in the dermis can provide innate protection without being primed by damaging compounds of the venom. 

Keratinocytes, which constitute 95% of epidermis cells and resident mast cells which are tissue-resident effector cells derived from different hematopoietic lineages, represent the first line of defense against venom-induced tissue injury. Mast cells can elicit de novo synthesis of proinflammatory mediators such as cytokines, chemokines, and eicosanoids leading to the attraction and activation of other effector immune cells [13,14,15]. Few studies have previously provided evidence that venom components can activate basal autophagy and proteasomal degradation in keratinocytes as part of constitutive immune responses [16]. Growing evidence suggests that mast cells and IgE have a critical role in the constitutive and innate immune responses to venoms [13]. In mice, adequate stimulation of mast cells prevents morbidity and mortality induced by diverse snake venoms, thanks to their release of cytokines and proteases degrading key components of the venoms [13,17,18]. For example, in the case of Russell’s viper venom, IgE and activation of mast cells can reduce the mortality of mice exposed to lethal amounts of venom [19].

### 3.3. Snake Venom Components Contributing to the Inflammatory Response

In the case of elapid and viper snake envenoming, components of the venoms, such as secretory phospholipases A2 (sPLA2) and snake venom metalloproteases (SVMP) in SVMP, directly contribute to local and systemic inflammatory responses [20,21,22,23]. 

Snake venom phospholipases A2 (sPLA2s) play a critical role in the initiation of inflammatory processes associated with snakebite envenoming. In venoms from Bothrops snakes, sPLA2s, i.e., the catalytically active Asp-49 enzymes and the catalytically inactive variant Lys-49, can evoke the classical inflammatory cascade eliciting local edema and leukocyte migration and adhesion in experimental animal models [24]. In rats, snake venom sPLA2s are able to induce mast cell degranulation and edematogenic activity. The proinflammatory effects of PLA2s have been attributed to the hydrolysis of membrane phospholipids, which generates a large number of lipid mediators such as leukotriene B4 (LTB4) displaying chemoattractant properties for leukocytes. The primary effect of svPLA2 is the production of arachidonic acid and its metabolites, which may activate the nuclear translocation of the transcription factor NFκΒ, which is responsible for the transcription of genes encoding cytokines (TNF-α, IL-1β, and IL-6) and phosphorylation of intracellular kinases (PI3K/Akt, MAPK p38, ERK1/2). In addition, arachidonic acid can be metabolized by cyclooxygenases and lipoxygenases, resulting in the release of pro-inflammatory molecules such as prostaglandins and leukotrienes [24,25,26]. 

Snake venom metalloproteases (SVMP) are involved in the hemorrhagic effects of venoms, owing to the loosening of connective tissue structures and degrading of blood vessel walls. Further evidence suggests that SVMP can initiate and support ongoing inflammation in experimental models in which injections of SVMP into rodents elicit increases in interleukins, PGE2, TNF-α, as well as modifications in leukocyte subtype repartition and migration capacities [22,27]. For example, batroxase, BaP1, and jararhagin snake venom metalloproteases can induce mast cell degranulation, release inflammatory mediators such as proinflammatory cytokines IL-1, IL-6, and TNF-α, activate the formation of complement components anaphylatoxins C3a, C4a, and C5a, and trigger leukocyte and macrophage infiltration [28,29]. 

The formation of blisters and local exudate are typical manifestations inflicted by venoms of snakes that have been studied to characterize the presence of an inflammatory response in the damaged tissues [30,31,32]. Previous proteomic studies of fluids collected from snake venom injured tissue has allowed identification of several mediators which are characteristic of on-going inflammatory response and tissue injury [31,32]. The most abundant proteins found in blister fluid exudates from bitten patients identified biological processes, such as platelet degranulation, inhibition of endopeptidase activity, innate immune responses, protease activities, endocytosis mediated by receptor binding, complement activation, disorganization of the extracellular matrix, and blood coagulation [31,32]. Of note, beside immunomodulators, blister fluids contain a wide variety of damage-associated molecular patterns (DAMPs), which are formed early in envenomation and can play a role in the activation of innate immune responses [32]. 

### 3.4. Innate Immune Responses in Snakebite Envenoming

Typically, the innate immune response primarily involves phagocytic cells and antigen-presenting cells (APCs), such as granulocytes, macrophages, and dendritic cells (DCs) [9,10,11]. The innate immune response is initiated following the recognition of evolutionarily conserved structures by a limited number of PRRs. These PRRs detect pathogen-associated molecular patterns (PAMPs), host-derived danger-associated molecular patterns (DAMPs), and molecular signatures associated with cytoplasmic homeostasis, which engage Toll-like receptors (TLRs). Major families of PRRs include Toll-like receptors (TLRs), retinoid acid-inducible gene I (RIG-I)-like receptors (RLRs), and nucleotide-binding oligomerization domain (NOD)-like receptors (NLRs). TLRs are mainly involved in antimicrobial and proinflammatory gene transcription. Downstream activities of PRR signaling elicit the synthesis of type I interferon (interferon-α (IFNα) and IFNβ), IL-1β, and tumor necrosis factor (TNFα). NOD1 and NOD2 are the best-characterized members of the NLR family, which result in pro-inflammatory gene transcription [9,10,11]. Distinct from these TLRs, other NLR proteins form a protein complex termed inflammasomes which can active inflammatory caspase-1, which catalyzes the cleavage of the IL-1β precursor to biologically active IL-1 β [33]. 

Growing evidence suggests a possible implication of damage-associated molecular pattern molecules (DAMPs) in the inflammatory response to snake venoms [34]. DAMPs can be present in skin blisters and released by damaged skeletal muscle [34,35,36]. A large variety of DAMPs have been identified, including, at least in part, cytochrome C and mitochondrial DNA, HMGB1, S100b, heat shock protein, fibrinogen, fibronectin fragments, and molecular pieces of extracellular matrix proteins [34,37]. Venom-induced DAMPs binding to cell signaling receptors such as TLR2, TLR4, and TLR9 receptors generate most of their biological activities via Toll-like receptor (TLR) engagement. Recognition of DAMPs by various TLRs is mediated by the MyD88 adaptor protein, which leads to the initiation of an intense inflammatory response [34,37,38,39,40,41].

### 3.5. Orchestration of the Inflammatory Response in Snakebite Envenoming

Once injected, toxins contained in the venom mixture can exert devastating local effects in the contiguous tissues and can be diffused systemically via the lymphatic system and blood vessels, leading to enabling toxins to act at various organ levels [1,2,3]. Nearly all venoms from viperid species and some elapid venoms induce local tissue injury characterized by an extensive local inflammatory process [1]. The involvement of the inflammatory process in the pathogenesis of snake envenomation has been reported since the 1990s. Many components of snake venoms (PLA2s, SVMPs, and SVSPs) contribute to local and systemic inflammatory processes, which is initiated by an increase of vascular permeability, followed by immune cell infiltration leading to the release of bioactive mediators [1,3,9,10]. Accordingly, the release of cytokines and chemokines, histamine, serotonin, bradykinin, platelet activating factor, prostaglandins, neurokinins, nitric oxide (NO), and anaphylatoxins (including the complement components C3 and C5), has been demonstrated in several experimental studies [9,10]. 

Another important characteristic of inflammation triggered by viperid snake venoms is the conspicuous infiltration of leukocytes, primarily neutrophils and then macrophages, into the local snakebite [11]. Leukocytes are essential cells in host defense thanks to the release of inflammatory mediators, such as COX1- and COX2-derived prostaglandins. The injection of snake venoms in animals induced leukocytosis with neutrophilia along with tissue infiltration at the site of injection. The role of leukocyte activation and tissue infiltration has been reviewed in much detail [9,10,11,42]. 

In brief, leukocyte accumulation in injured tissues induces the production of chemotactic factors concurring to the adhesive interactions between leukocytes and endothelial cells within the microcirculation. Leukocyte recruitment into the bitten sites is known to be a consecutive series of events characterized by an initial interaction of leukocytes with the endothelium, or rolling, followed by leukocyte firm adhesion and diapedesis through blood vessels [43,44]. Leukocyte rolling along the endothelium is mediated by the selectin family (L-selectin on neutrophils, and P-selectin and E-selectin on endothelial cells). Next, endothelial intercellular adhesion molecule-1 (ICAM-1), a member the immunoglobulin superfamily, binds to leukocyte-function-associated antigen-1 (LFA-1) (CD11/CD18) [43,44]. Leukocyte–endothelium interactions provoke firm adhesion that is required for trans-endothelial cell migration, or diapedesis. Finally, platelet endothelial cell adhesion molecule-1 (PECAM-1) is implicated in the mechanisms leading to the passage of leukocytes through endothelial junctions and/or the basement membrane [43,44]. Experimental studies carried out by injecting snake venoms in animals have reproduced the leukocytosis with neutrophilia observed in some clinical cases. Experimental studies suggest that ICAM-1, LECAM-1, LFA-1, and PECAM-1 adhesion molecules are involved in the recruitment of neutrophils into the inflammatory site induced by snakebite. Mounting in vivo evidence has shown that administration of snake venom up-regulates the expression of leukocytes and endothelial cell adhesion molecules [42,45]. The production of bioactive molecules by leukocytes, such as ROS and proteinases, as well as the formation of neutrophil extracellular traps (NETs), are involved in the mechanisms of local tissue injuries following administration of snake venoms [42]. 

## 4. Snakebite-Induced Oxidative Stress

Another important feature of the pathogenesis of snake envenomation is the production of reactive oxygen species (ROS). Typically, the generation of ROS can potentiate tissue damage at inflammatory sites following snakebite. Whereas previous studies have witnessed its prevalence, the involvement of oxidative stress in envenomed patients remains an unrecognized scenario [46,47]. Most of the available studies have used either crude venom preparations or specific venom components to test their pro-oxidant potential. 

### 4.1. Oxidative Stress Elicited by Crude Venom

The involvement of oxidative stress in the pathogenesis of snake envenomation was initially reported in the early 1980s [48,49]. Intraperitoneal (IP) injection of Russell’s viper venom in adult mice can elicit a rapid rise of malonaldehyde produced by the liver, heart, lung, kidney, and brain, suggesting endogenous lipid peroxidation [48]. Consistently, IP injection of *Echis pyramidum* viperid venom in adult mice induced an early and prolonged increase in lipid peroxidation [50]. In this later study, the onset of oxidative stress was as early as 1 hour and lasted for several hours. IP injection of *Crotalus durissus terrificus* viperid venom increased levels of oxidized glutathione/reduced glutathione ratio GSSG/GSH ratio in the cortex of kidney and renal medulla of mice [51]. Similarly, IP injection of *Crotalus durissus terrificus* viperid venom in rats caused increases of liver lipid peroxidation, catalase, and glutathione S-transferase (GST) activity, while hepatic levels of reduced glutathione (GSH) were unchanged [52]. The injection of elapid venoms of the Egyptian cobra (*Naja haje)* also induced increases in serum levels of malonaldehyde, while serum levels of GSH, superoxide dismutase (SOD), and catalase (CAT) were consistently reduced [53]. Overall, these results suggest that oxidative stress may occur in experimental in vivo envenomation and may be responsible, at least in part, for hepatic and renal damage in both viperid and elapid species. In contrast with these deleterious effects, it has been demonstrated that generation of ROS by snake venom may have anticancer activities, which include the inhibition of cancer cell proliferation, cell cycle arrest, and induction of apoptosis [54,55]. Components of the snake venom mixture, such as L-amino acid oxidase, phospholipase A_2_, lectin, and disintegrins, have the potential to stimulate the production of ROS of cancer cell lines leading to alteration of cell motility, cell invasion potential, and apoptosis [54,55,56,57,58]. Growing evidence suggests that several toxins of the snake venom mixture can modulate key cancer-related features such as ROS-dependent DNA damage, extracellular matrix–integrin signaling inhibition, cytoskeleton network disruption, growth factor inhibition, and mitochondrial dysfunction. This offers the hope to test new anticancer drugs based on toxin motifs. Evaluation of efficacy, potency, and safety of such toxin-derived scaffolds are challenging but are needed to establish future in vivo applications.

Oxidative stress induced by snake venoms has also been related to tissue sequestration of activated leukocytes. As a part of host defense against offending agents, leukocyte priming at the snakebite site has been described in animal models of viperid envenomation. Injection of *Bothrops asper* and *Bothrops jararaca* venoms can elicit a long-lasting infiltration of leukocytes when venom is injected into the peritoneal cavity of mice [59]. In this study, H_2_O_2_ production by leukocytes harvested from the peritoneal cavity after *Bothrops* venom IP injection was significantly increased. In agreement with this finding, venoms from viperids and elapids (*Echis carinatus* and *Naja naja*) have been shown to elicit superoxide generation, H_2_O_2_ production, and NADPH oxidase activation in rodent and human in vitro models [42,60,61,62]. For example, human blood incubated with Russell’s viper venom can stimulate the production of H_2_O_2_ and malondialdehyde, along with the increase of superoxide dismutase (SOD) levels [63]. 

In vivo, systemic oxidative stress was further evaluated in human victims of *Bothrops* snakebites monitored at the hospital for up to 30 days [64]. In this clinical study, initial determinations at the emergency entrance revealed increased catalase, glutathione reductase, and glutathione peroxidase activities, along with decreased SOD and GST activities and depletion of GSH contents. In this series of snakebite victims, markers of oxidative damage included increased malondialdehyde levels compared with controls blood donors. In human envenomation, growing evidence suggests that oxidative stress can play a critical role in the pathophysiology of snakebite, predominantly from viperids.

### 4.2. Oxidative Stress Induced by Specific Snake Venom Components

Phospholipases A_2_, metalloproteinases, three-finger toxins (3FTx), and L-amino acid oxidase are known to induce oxidative stress [46]. 

PLA2s are major components in the proteome analysis of viper venom and these lipolytic enzymes are known to induce tissue necrosis and the inflammatory cascade [1,6,8,65]. PLA2s elicit the phospholipid ester bond cleavage at the sn-2 site by nucleophilic attack. On the other hand, calcium can stabilize the negatively charged transition state by allowing the coordination of phosphate oxygen and a carbonyl group during the process of catalysis. It is believed that PLA2s alter membrane fluidity and favor membrane permeabilization [66]. Hence, that PLA2s induce necrosis is mainly attributed to their action on membrane lipid degradation, further contributing to oxidative stress. In particular, destruction of the plasma membrane of the cell favors Ca^++^ influx, which in turn activates mitochondrial ROS generation by impairing the function of the electron transport chain [67]. Calcium-dependent activation of PLA2s elicit an increase in mitochondrial eicosanoids from arachidonic acid, which interacts with complex I of the electron transport chain to increase superoxide and, subsequently, H_2_O_2_ generation [67,68]. Ca^++^ influx can activate the Ca^++^-dependent proteases of the cytosol, such as calpains and lipoxygenases, which are also involved in the disruption of cellular integrity. Cytosolic Ca^++^-dependent proteases can damage protein structures or the lipid bilayer, thereby leading to necrotic cell death [69,70]. 

Mitochondrial ROS production has also been described in cellular models using beta-neurotoxins, such as the neurotoxic phospholipase A_2_. Beta-neurotoxins initiate neuromuscular blockade via pre-synaptic binding of the motor nerve ends, eliciting the depletion of synaptic acetylcholine (ACh) vesicles, altered ACh release, and later on, motor nerve terminal destruction [71]. In this context, beta-bungarotoxin of krait (genus *Bungarus*) belonging to the elapid cobra family has been shown to induce neuronal inactivity and significant mitochondrial ROS production [72]. These events are related to initial PLA_2_ phospholipid enzymatic hydrolysis at the plasma membrane level of nerve ends. Lysophospholipid and fatty acid release induces biophysical changes within the cell membrane leading the synaptic vesicles to membrane fusion and to exocytosis of the vesicle pool. In addition, ion permeability is increased, leading to membrane depolarization and calcium entry, which favor exocytosis of the reserve pool of vesicles. Overall, presynaptic vesicles are depleted along with mitochondrial alterations, including major mitochondrial oxidative stress. 

Viperid venom mixture contains high amounts of snake venom metalloproteinases (SVMPs), while are present in lower amounts in elapid venoms [1,6,8]. SVMPs represent a wide group of multidomain proteins displaying several biological activities which may elicit hemorrhage, myonecrosis, degradation of fibrinogen and extracellular matrix (ECM), inflammation, and inhibition of platelet aggregation [1,6,8]. Hemorrhagic SVMPs cleave proteins of ECM, especially at the membrane basement that provides mechanical stability to micro-vessels. Rupture of micro-vessel walls induces hemorrhage, which can contribute to red blood cell extravasation and hemolysis associated with the release of hemoglobin and redox-active free iron triggering oxidative stress in target organs [73]. Likewise, SVMPs can induce muscle necrosis and myoglobin release altering redox balance.

L-amino acid oxidase (LAAO) is a flavoenzyme catalyzing the oxidative deamination of L-amino acid to α-keto acid and producing hydrogen peroxide (H_2_O_2_) [6]. Although LAAO is not present in large amounts in snake venom mixture, it is prevalent in many snake venoms [8,70]. LAAO can elicit edema formation, hemolysis, platelet aggregation, and toxicity in myocytes [1,6,8,70]. Most of the pro-oxidant effects of LAAO are related to H_2_O_2_ generation along with its catalytic activity [74,75,76]. In line, LAAO exposure to glutathione (GSH) or catalase can reduce LAAO-induced H_2_O_2_ production [76,77,78]. Cytotoxicity of LAAO has been related to activation of the apoptotic machinery, which tightly regulated ROS production [70]. 

Elapid snake venom mixture contains high amounts of three-finger toxins (3FTxs [1,6,8]. 3FTxs are non-enzymatic proteins, which include acetylcholinesterase inhibitors, antiplatelet active molecules, L-type calcium blockers, type IA and IB cytotoxins (CTXs), types I, II, and III α-neurotoxins, and muscarinic toxins [6]. Neurotoxins active at the post-synaptic site (alpha-neurotoxins) bind to the post-synaptic nicotinic ACh receptors of the muscle. The action of these neurotoxins mimics the action of d-tubocurarine; they are therefore designated as ‘‘curare-mimetic’’ toxins. They produce a reversible, non-depolarizing block at the post-synaptic site by competitive inhibition of ACh binding to the nicotinic ACh receptors [6,71]. It has been recently reported that synaptic inactivity induced by 3FTx muscle (alpha-bungarotoxin) or 3FTx nerve (alpha-latrotoxin) neurotoxins can significantly increase mitochondrial ROS production in vivo [72]. 

Snake cytotoxins, also called cardiotoxins (CTXs), are 3FTxs that consist of about 60 amino acid residues and shed four disulfide bonds [79]. CTXs isoforms are found in many cobras, as well as rattlesnake venom [1,2,3]. The commonly accepted mechanism of CTXs consists of the arrangement of non-bilayer immobilized toroidal structures within the membrane, which is caused by the translocation of CTXs into the intermembrane space. While CTXs are highly basic amphipathic proteins, CTXs can selectively focus on mitochondria by interacting with anionic phospholipids such as cardiolipin in inner mitochondrial membranes. The localization of CTXs into mitochondria can promote fragmentation of the mitochondrial network, mitochondrial density reduction, decrease of the basal mitochondrial oxidative phosphorylation, and oxidative stress [80,81,82,83,84]. 

## 5. Long-Term Sequelae Secondary to Snakebite Envenoming

Although acute renal failure, organ necrosis, bleeding, neuro/cardio toxicity, and myonecrosis are well known acute damaging effects of snakebite envenoming, long-term effects following snakebite have not been extensively evaluated [1,2,3]. The most frequent delayed complications observed in patients with snake envenomation on long-term follow-up are ulceration, amputation, renal failure, and musculoskeletal disabilities [85].

Infirmity due to amputations, deformities, muscle contractures, and chronic leg ulcers can be seen after local necrosis induced by snakebites mainly from African and Asian cobras and Latin American pit-vipers [85]. When venoms cause myotoxicity without affecting the vasculature, such as those of many elapid snakes, the muscle regeneration is processed adequately. On the other hand, in envenoming related to viperid snakes, which destroy the vascular walls and the extracellular matrix in addition to myocyte structures, muscle regeneration is dramatically impaired. In the case of viperid venoms, muscle mass is therefore reduced and replaced by fibro-adipose tissue. Hence, skeletal muscle regeneration following snake venom-induced myonecrosis largely depends on the action of snake toxins on specific targets [86]. Acute muscle damage, i.e., myonecrosis, is related to the activities of venom PLA2s and cardiotoxins (CTXs) present in elapid and viperid snake venoms. The toxins that do not destroy the myofiber’s basal lamina and blood vessel walls can result in successful regeneration, as regeneration occurs within the old basal lamina [86,87,88]. CTX induces early-onset inflammatory cell recruitment and prominent oxidative stress, which results in the activation of muscle satellite precursor cells, [89]. Viperid venoms contain hemorrhagic SVMPs, which also contribute to muscle necrosis by generating ischemia and hypoxia in the tissue. In this setting, hypoxia is considered a consequence of ample extravasation of plasma fluid due to the secondary destruction of capillary blood vessel walls by the enzymatic action of SVMPs [86]. Here, components of venom damage can destroy blood vessels, nerves, and basal lamina, resulting in impaired muscle regeneration [86,90,91]. Growing evidence suggests the implication of reactive oxygen species (ROS) in the cellular and molecular processes taking place during the complex process of muscle regeneration [92]. Hence, it is warranted to decipher and target ROS networks to provide precise insight of the redox regulation of skeletal muscle regeneration after snake-venom-induced myonecrosis.

Viperid and some elapid snakebite envenoming can induce acute renal injury. Proposed mechanisms include ischemia secondary to changes in renal blood flow secondary to systemic bleeding, vascular leakage, and hypotension. Depending on venom composition, proteolytic destruction at the glomerular basement membrane site, formation of microthrombi in the renal microvasculature, direct cytotoxic action of venom components such as PLA2s on renal tubular cells, and myoglobin accumulation in renal tubules along with rhabdomyolysis may occur [93]. Acute renal injury is reversible thanks to treatment with antivenom and supportive care; however, its progression into chronic renal failure has been reported in some cases following pit viper bites. In line with studies showing that the kidney is highly vulnerable to oxidative stress, direct and indirect nephrotoxic actions of venom components can impair redox balance leading to irreversible oxidative damage to the kidneys. Consistently, experimental studies confirmed the direct toxicity of *Bothrops* venom on renal epithelial cells related to the excessive generation of reactive oxygen species (ROS), dissipation of mitochondrial membrane potential, caspase activation, and apoptosis [94,95]. In rodent studies, the deleterious role of oxidative stress induced by snake venom has also been described [96,97]. In human envenomation, oxidative stress has been implicated in the pathogenesis of snakebite-induced renal failure and damage [98,99].

## 6. Therapeutic Perspectives Using Antioxidant Molecules

Although snakebite envenomation is a multifactorial insult, administration of antivenoms is the only prescribed treatment. While antivenom directly inhibits venom toxins, it has limitations to treat snakebite-induced local effects and late onset complications. The use of antioxidant molecules such as N-acetyl-cysteine and allopurinol has provided some beneficial effects in experimental models of snake envenomation [100,101]. In addition, compounds isolated from plants have been tested in various preclinical models of envenomation thanks to their antioxidant properties. The inhibitory effects of plant extracts have been evaluated either by applying practices followed in local communities or by using specific active principles. Among antioxidant natural extracts, flavonoids have consistently demonstrated inhibitor effects on snake venom activities both in in vitro models and in preclinical experiments. Interestingly, quercetin and its glycosides are natural plant flavonoids that have been studied for their antioxidant properties to treat snake envenomation. Glycosylated quercetin forms were first shown to inhibit snake venom PLA2s in vitro [102,103]. Preclinical studies have eventually shown that quercetin-3-O-rhamnoside and quercetin-3-rutinoside (rutin) had an inhibition activity on edema, hemorrhage, and lethality in a mouse model [103,104,105]. The action of these compounds was then demonstrated to occur at the cytosolic cell level and involved the direct trapping/neutralization of ROS and inhibition of oxidative-stress-induced side effects [105]. However, despite abundant research in the field of natural antioxidant compounds and snake envenomation, there is no naturally derived drug approved for clinical use in the treatment of snakebite envenoming [106].

## 7. Conclusions

Mounting information suggests that oxidative stress is a key player involved in the pathophysiology of snakebite envenomation. Following venom administration in the body, enzymatic and non-enzymatic components of the venom mixture induce deleterious actions that impair redox homeostasis. Venom-induced oxidative stress participates in local and systemic complications in the initial stages of envenomation. In addition, oxidative stress elicited by snakebite envenomation can play a critical role in the development of secondary/ long-term complications such as renal failure and persistent local tissue degradation and musculoskeletal infirmities. 

## Data Availability

Not applicable.
